# Colonic perforation resulting from ingested chicken bone revealing previously undiagnosed colonic adenocarcinoma: report of a case and review of literature

**DOI:** 10.1186/1477-7819-9-24

**Published:** 2011-02-18

**Authors:** Douglas H McGregor, Xiaoying Liu, Ozlem Ulusarac, Kimberly D Ponnuru, Stephanie L Schnepp

**Affiliations:** 1 Department of Pathology and Laboratory Medicine, University of Kansas Medical Center, Kansas City, Kansas, USA; 2 Pathology and Laboratory Medicine Service, Veterans Affairs Medical Center, Kansas City, Missouri, USA; 3 Surgical Care Service, Veterans Affairs Medical Center, Kansas City, Missouri, USA; 4 Department of Surgery, University of Missouri - Kansas City, Kansas City, Missouri, USA

## Abstract

An 86 year old male with a four-day history of nonspecific gastrointestinal symptoms was found on colonoscopy to have evidence of sigmoid colon obstruction and possible perforation. Emergent operative exploration revealed diffuse peritonitis, sigmoid perforation, adjacent dense adhesions, and a foreign body protruding through the perforated area. Pathologic examination showed the foreign body to be a sliver of bone consistent with chicken bone and the sigmoid subacute perforation to be associated distally with a circumferential ulcerated obstructing mass, microscopically seen to be transmurally infiltrating adenocarcinoma, signet-ring cell type. There was extensive acute and organizing peritonitis, 100% Escherichia coli was cultured from peritoneal fluid, and the patient died two days postoperatively with sepsis and hypotension. This appears to be the fifth reported case of colonic perforation resulting from foreign body perforation due to previously undiagnosed adenocarcinoma. The four previously reported cases were all deeply invasive adenocarcinoma of sigmoid colon, and the foreign bodies included three chicken/poultry bones and a metallic staple. These five cases are highly unusual examples of a potentially lethal malignant neoplasm being clinically revealed by a usually (but not always) innocuous event, the ingestion of a small foreign body.

## Background

Colonic perforation is most often secondary to extrinsic or intrinsic obstruction, but occasionally it may be due to other factors such as foreign bodies. Over 300 cases of bowel perforation caused by foreign bodies have been reported in the literature, with fish bones, chicken bones and dentures being the commonest objects, followed by toothpicks and cocktail sticks [1]. Foreign body-associated perforation commonly occurs at the point of acute angulation and narrowing, and the most common site of perforation is the terminal ileum and colon, with an increased number of reports of perforation in association with Meckel's diverticulum, the appendix and diverticular disease [2,3].

Symptoms related to obstructing colon cancer are often delayed, and the present reported case is an interesting example of ingested foreign body resulting in both colon perforation and the discovery and resection of a previously undiagnosed colon cancer. This case appears to be the fifth reported example of colon perforation resulting from foreign body perforation due to previously undiagnosed colon cancer [4-7]. Table 1 outlines the basic specifics of these five cases. Not surprisingly, all of these obstructing colon cancers were large deeply invasive adenocarcinomas and their locations were the anatomically distal and relatively narrow sigmoid colon. The foreign bodies included 4 chicken/poultry bones (as in the present case 5), and a metallic staple. The clinical outcomes were full recovery (cases 1 and 2), postoperative death due to sepsis (case 5) and unknown (cases 3 and 4).

**Table 1:** Reported cases of colon perforation resulting from foreign body and previously undiagnosed carcinoma

**Case No**.	Age/Sex	Colon Site	Carcinoma Morphology	Foreign Body	Outcome	Reference No/Year
1	78/F	Sigmoid	Large carcinoma	Chicken bone	Full recovery	4/1985

2	64	Sigmoid	6.5 cm long circular ulcerated moderately differentiated adenocarcinoma without stenosis pT3 N0 M0	Poultry bone	Full recovery	5/1996

3	57/M	Sigmoid	Exophytic infiltrating moderately differentiated adenocarcinoma pT4 N1 M0	Metallic staple	Unknown	6/1997

4	69/M	Sigmoid	Polypoid mass, adenocarcinoma	Chicken bone	Unknown	7/2001

5	86/M	Sigmoid	5.5 × 4.4 cm circumferential ulcerated mucinous/signet ring adenocarcinoma pT3 N2 MX	Chicken bone	Died 2 days postop from sepsis	2010

## Case report

An 86 year old male presented with a four-day history of abdominal pain, nausea, vomiting, and intolerance to oral intake. Physical exam demonstrated left lower, right lower and left upper quadrant tenderness, but clinical evidence of colonic obstruction and acute abdomen was not identified. Vital signs were temperature 98.5, pulse 86, respiration 20 and blood pressure 136/62. Laboratory data included WBC 6.4 K/cmm, neutrophils 87.5%, hemoglobin 11.5 g/dl and hematocrit 37.4%. Radiologic abdominal exam demonstrated a normal gas pattern, and ultrasound and CT scan studies were not indicated. Colonoscopy (preceded by midazolam and demarol medication) was performed for evaluation of iron deficiency, however, and showed evidence of sigmoid colon obstruction and possible perforation, including a mass with narrowing at 30 cm and a cavernous defect with whitish exudate. The patient underwent emergent operative exploration, which revealed diffuse peritonitis, a sigmoid perforation, adjacent dense adhesions, and a foreign body protruding through the perforated area. Sigmoid colon resection and end colostomy with Hartman's pouch was performed.

**Figure 1:**
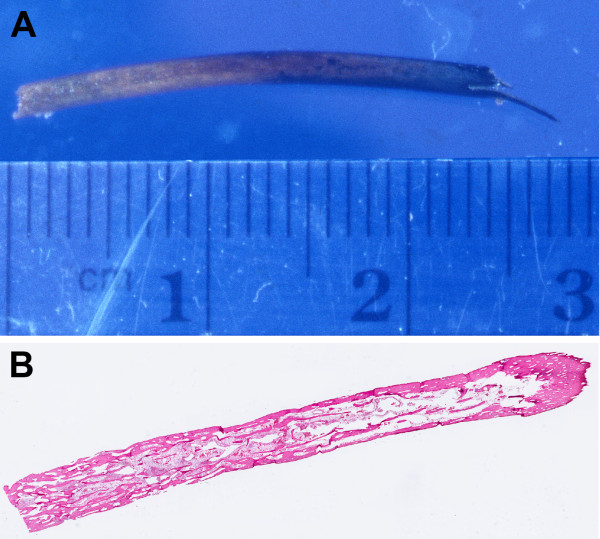
**Foreign body, found intraoperatively to be protruding through the colonic perforation**. (A) Gross, consistent with sliver of bone, (B) Microscopic, confirming the boney nature of the foreign body.

**Figure 2:**
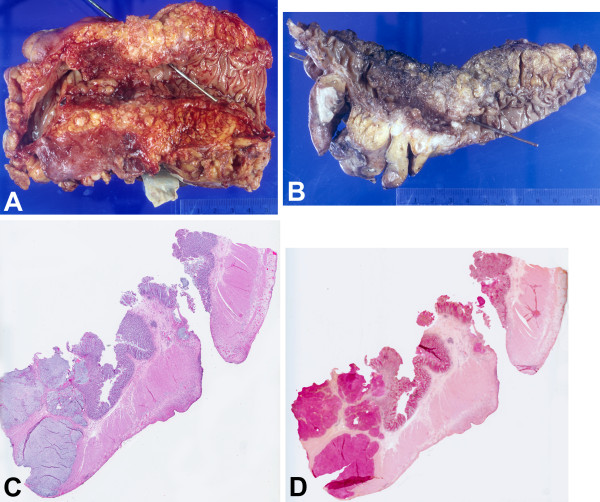
**Segment of colon**. (A) Gross, with probe through site of perforation and obstructing ulcerated mass to the left of (distal to) the perforation, (B) Gross, with longitudinally sectioned colon showing relationship between the perforation (with probe) on the right and the obstructing ulcerated mass on the left, (C) Microscopic, with the proximal perforation on the right and the distal transmurally invasive adenocarcinoma on the left (H&E, 1×), (D) Microscopic, same section as (C), showing the mucinous nature of the carcinoma (mucicarmine, 1×) 14.

Specimens received for pathologic examination included the foreign body, segment of sigmoid colon, and additional segment of sigmoid colon. The foreign body, which had been found to be protruding through the perforation, consisted of a sliver of bone measuring 2.6 × 0.2 cm (Figure 1A) and the boney nature of this foreign body was confirmed microscopically (Figure 1B). The segment of sigmoid colon had a 5.5 × 4.4 cm circumferential ulcerated mass with marked luminal obstruction and a 0.2 × 0.2 cm perforation 1.0 cm proximal to the mass. (Figure 2A, B)

Microscopically, the colonic mass distal to the perforation, was a poorly differentiated adenocarcinoma, signet ring cell type (histologic grade 4), with invasion through the muscularis propria into subserosal adipose tissue (Figure 2C, D), and there were metastases in 20 of 35 pericolic lymph nodes (pathologic stage T3 N2 MX). The colonic perforation was found to be subacute, with extensive acute and organizing peritonitis. 100% heavy growth of Escherichia coli was cultured from peritoneal fluid. Postoperatively, the patient remained septic and hypotensive, and he expired two days later.

## Conclusions

Colonic perforation is usually due to extrinsic or intrinsic obstruction, but occasionally other factors such as foreign bodies may be involved. We report here a case of sigmoid colon perforation which resulted from an ingested chicken bone penetrating the colonic wall due to obstruction by a previously undiagnosed sigmoid colonic adenocarcinoma. This appears to be the fifth reported case of colonic perforation resulting from foreign body perforation due to previously undiagnosed adenocarcinoma.

Table 1 outlines the basic specifics of these five cases. Not surprisingly, all of these obstructing colon cancers were large deeply invasive adenocarcinomas and their locations were the anatomically distal and relatively narrow sigmoid colon. The foreign bodies included 3 chicken/poultry bones (as in the present case 5) and a metallic staple. The clinical outcomes were full recovery (cases 1 and 2), postoperative death due to sepsis (case 5) and unknown (cases 3 and 4).

The above case report and four previous cases show the similarities among these five cases - highly unusual examples of a potentially lethal malignant neoplasm being clinically revealed by a usually (but not always) innocuous event, the ingestion of a small foreign body.

## Consent

Written informed consent was obtained from the patient's next of kin for publication of this case report and any accompanying images. A copy of the written consent is available for review by the Editor-in-Chief of this journal.

## Competing interests

The authors declare that they have no competing interests.

## Authors' contributions

DHM and XL conceived the idea of the manuscript, conducted a literature search and drafted the manuscript. OU edited the manuscript and assisted in the submission process. KDP and SLS performed the sigmoid segmental resection.

## Author's information

Douglas H. McGregor is Professor of Pathology at the University of Kansas Medical Center and Director of Surgical Pathology at the Kansas City Veterans Affairs Medical Center, and he has been a manuscript reviewer for the World Journal of Surgical Oncology. Xiaoying Liu was Pathology Resident and Cytopathology Fellow at the University of Kansas Medical Center when this manuscript was conceived and developed, and she is currently Assistant Professor at Dartmouth-Hitchcock Medical Center, Lebanon, New Hampshire. Ozlem Ulusarac is Assistant Professor of Pathology at the University of Kansas Medical Center and Director of Microbiology/Immunology and Chemistry at the Kansas City Veterans Affairs Medical Center. Kimberly D. Ponnuru is Assistant Clinical Professor of Surgery at the University of Missouri - Kansas City and Staff Surgeon at the Kansas City Veterans Affairs Medical Center. Stephanie L. Schnepp was Surgery Resident at the University of Missouri - Kansas City at the time of the patient's surgery and currently practices general surgery with Bellevue Surgical Associates, Saint Louis, Missouri.
